# Merkel cell carcinoma with in-transit metastases: Complete response after intralesional interleukin-2 therapy

**DOI:** 10.1016/j.jdcr.2026.04.026

**Published:** 2026-04-21

**Authors:** Andrés Vidal González, Sergio López Alcázar, Rosa Feltes Ochoa, Rocío Gil Redondo, Pedro Herranz Pinto, Ander Mayor Ibarguren

**Affiliations:** Department of Dermatology, Hospital Universitario La Paz, Madrid, Spain

**Keywords:** immunotherapy, interleukin-2, Merkel cell carcinoma

## Introduction

Merkel cell carcinoma (MCC) is a rare cutaneous neuroendocrine malignancy characterized by aggressive behavior and high rates of recurrence and mortality. Its incidence has increased in recent decades, particularly among elderly and immunosuppressed individuals.^1^ Two main, nonmutually exclusive mechanisms are involved in its pathogenesis: integration of Merkel cell polyomavirus and cumulative ultraviolet radiation–induced DNA damage.^2^

The treatment of localized MCC is based on surgical excision with adequate margins, often combined with adjuvant radiotherapy. According to recent European consensus recommendations, sentinel lymph node biopsy is recommended in all patients with MCC without clinically detectable lymph node involvement or distant metastases.^1^^,^^3^ In advanced stages, the introduction of immune checkpoint inhibitors has significantly transformed the therapeutic management of MCC. Both avelumab and pembrolizumab have demonstrated durable responses and acceptable safety profiles, becoming first-line treatment options compared with conventional chemotherapy.^4^^,^^5^

Despite these advances, a proportion of patients may experience disease progression during immunotherapy. In this context, locoregional therapies gain relevance for the control of cutaneous or subcutaneous disease and may represent a useful alternative in selected cases. Locoregional treatment options include palliative radiotherapy, electrochemotherapy, isolated limb perfusion or infusion, and, in a more experimental setting, intralesional therapies.^6^, ^7^, ^8^, ^9^

## Case presentation

An 80-year-old man with a medical history of dyslipidemia and ischemic heart disease was followed in the Dermatology Department for multifocal MCC on the medial aspect of the left lower leg (T2*N*2M0, stage IIIB according to the AJCC eighth edition). A complete response was achieved following radical radiotherapy and adjuvant avelumab. Fifteen months after diagnosis, while receiving treatment with avelumab, he developed disease progression with the appearance of retroperitoneal lymphadenopathy and in-transit metastases in the same limb, which were histologically confirmed. Lymph node radiotherapy and electrochemotherapy with bleomycin were added to his treatment.

**Fig 1:**
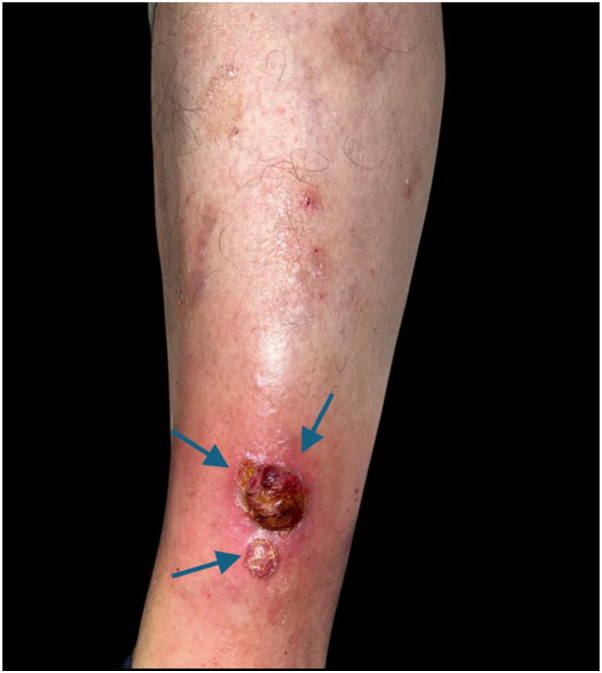
Erythematous–violaceous nodules on the lateral aspect of the left lower leg, consistent with in-transit metastases of Merkel cell carcinoma before intralesional IL-2 therapy

Following a partial response to electrochemotherapy with bleomycin, with persistence of 3 exophytic lesions despite continued avelumab therapy (Fig 1), a multidisciplinary tumor board decided to add intralesional interleukin-2 therapy (aldesleukin, 3 million IU, twice weekly) combined with cryotherapy. The dosing strategy was based on previously described protocols for intralesional IL-2 therapy.^10^^,^^11^ Following the recommendations of Garbe et al, the injected volume was adjusted according to lesion size (<5 mm: 0.2 mL; 5-10 mm: 0.4 mL; 10-20 mm: 1 mL; >20 mm: up to 2 mL), with the dose distributed among lesions. In our patient, approximately 3 million IU of aldesleukin were administered per session twice weekly using intratumoral injections with an insulin-type syringe. Cryotherapy was applied immediately after intralesional IL-2 administration. This approach was used to induce tumor cell disruption and antigen release, potentially enhancing the local immune response stimulated by IL-2.^12^

**Fig 2:**
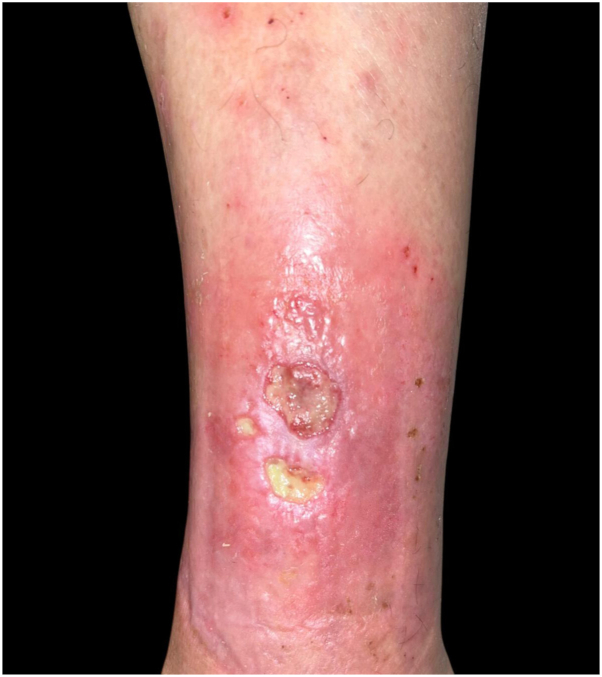
Complete ulceration of the tumor lesions after 2 mo of intralesional IL-2 treatment. Surrounding erythematovesicular plaques consistent with eczematous dermatitis.

**Fig 3:**
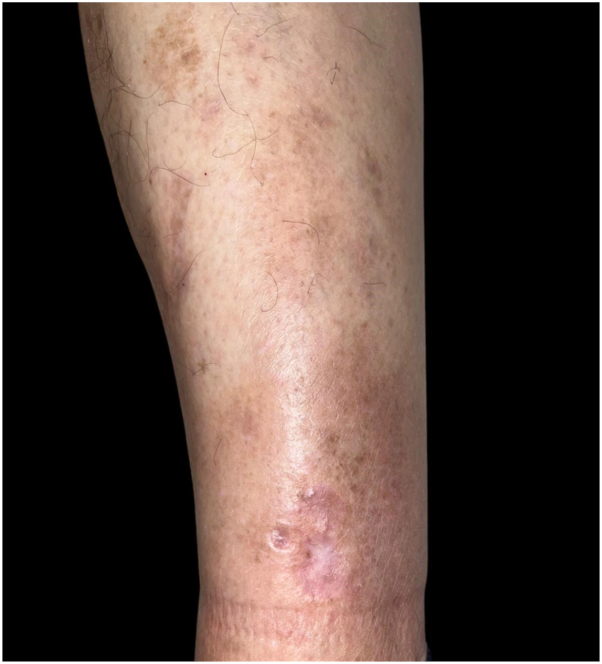
Complete re-epithelialization of previous ulcers with no signs of tumor recurrence after 3 mo of intralesional IL-2 treatment.

After 7 treatment sessions, the patient developed ulceration and necrosis of the treated lesions, accompanied by perilesional eczematous changes (grade 2, CTCAE v5.0) (Fig 2). Three months after initiation of therapy, a complete clinical response of the cutaneous lesions was observed (Fig 3). Subsequent imaging revealed progression of popliteal and inguinal lymph nodes, with tumor infiltration confirmed by core needle biopsy. The patient was therefore enrolled in a clinical trial with PM-14 plus atezolizumab.

During participation in the clinical trial, the cutaneous response was maintained; however, due to inguinal nodal progression, the patient discontinued the trial and received radiotherapy, achieving a partial radiological response that is currently maintained. A timeline summarizing treatments and clinical evolution is shown in Table I. After 2 years of follow-up, no local cutaneous progression has been observed, and the complete response achieved with intralesional IL-2 has persisted.

**Table I:** Timeline of treatments and clinical course of the patient

Time point	Clinical course	Treatment
Initial diagnosis	Multifocal Merkel cell carcinoma diagnosed on the left lower leg (T2*N*2M0)	Radical radiotherapy + adjuvant avelumab.
+ 15 mo	Development of in-transit metastases on the left lower leg and retroperitoneal lymphadenopathy	ECT with bleomycin (skin) + radiotherapy (retroperitoneum) + avelumab
+ 18 mo	Persistence and progression of cutaneous metastatic lesions	Intralesional IL-2 initiated twice weekly in combination with cryotherapy + avelumab
+19 mo	Progressive reduction in size and inflammation of lesions	Ongoing intralesional IL-2 therapy + avelumab
+ 21 mo	Complete clinical response of the cutaneous lesions with full re-epithelialization.	IL-2 therapy discontinued after 7 injections + avelumab
+ 25 mo	Nodal progression involving the inguinal and popliteal lymph nodes. No skin lesions.	Enrollment in a clinical trial evaluating PM-14 in combination with atezolizumab (PM14-A-003-20)
+45 mo	Nodal progression involving the inguinal lymph nodes	Discontinuation from the clinical trial, retreatment with radiotherapy

*ECT,* Electrochemotherapy.

## Discussion

We report a patient with multifocal MCC of the left lower limb with in-transit metastases, refractory to treatment with avelumab, and electrochemotherapy with bleomycin. After multidisciplinary evaluation, intralesional IL-2 therapy (3 million IU, twice weekly) was initiated, resulting in a complete cutaneous response after 3 mo, without relevant systemic adverse effects. Subsequent nodal progression was observed, but no cutaneous recurrence occurred.

This case illustrates the potential utility of local immunomodulatory therapies for regional disease control. Intralesional IL-2 has demonstrated efficacy in melanoma with in-transit metastases, with complete response rates of 50% to 70% and durable responses exceeding 12 mo in most patients.^13^^,^^14^ In published series, complete responses were maintained for more than 12 m in the majority of patients.^13^

Interest in IL-2–based immunotherapy in MCC has also been explored in the IMMOMEC trial, which investigated targeted delivery of interleukin-2 to the tumor microenvironment.^15^ Although the rapid development of immune checkpoint inhibitors subsequently transformed the therapeutic landscape of MCC, this project highlighted the potential role of IL-2–based approaches in stimulating antitumor immune responses.

Although experience with intralesional IL-2 in MCC is limited, complete local responses have been described with other intralesional therapies, such as talimogene laherparepvec or tavokinogene telseplasmid (IL-12 delivered by electroporation), in refractory cutaneous lesions. However, talimogene laherparepvec was not available at our institution at the time of treatment. These findings suggest a potential role for this strategy as a bridging or adjuvant therapy in patients who fail systemic immunotherapy.^16^^,^^17^ The systematic review by Raeber et al further supports the immunologic rationale and favorable safety profile of intralesional therapies, highlighting their potential synergy with immune checkpoint inhibitors.^17^

While systemic immunotherapy remains the cornerstone of treatment for advanced locoregional or metastatic MCC, increasing evidence supports the consideration of local treatments—such as radiotherapy, electrochemotherapy, or intralesional immunotherapy—in selected patients with cutaneous recurrences or disease confined to the skin, always within a multidisciplinary decision-making framework. In this setting, intralesional IL-2 may contribute to local disease control and reduction of tumor burden while preserving quality of life. To the best of our knowledge, this represents the first reported case of successful treatment of Merkel cell carcinoma with in-transit metastases using intralesional IL-2.

The combination of systemic immunotherapy with local strategies such as electrochemotherapy or intralesional IL-2 represents a promising area of research, with potential immunological synergy and a low additional risk of toxicity. In patients with cutaneous progression under PD-L1 inhibitors, this approach may help re-stimulate antitumor immune responses without significantly increasing toxicity.

In summary, intralesional IL-2 may represent a reasonable therapeutic option for local control of refractory cutaneous MCC, with good tolerability, potential for complete response, and a solid immunological rationale. Although these findings should be interpreted with caution, as they are based on a single case, further studies are required to better define its role.

## Conclusion

Intralesional IL-2 may be considered an effective and well-tolerated local therapeutic option in refractory MCC, particularly in patients with disease confined to the skin or subcutaneous tissue. Its use should be individualized within a multidisciplinary setting, as a complementary or bridging strategy following failure of systemic immunotherapy.

## Conflicts of interest

None disclosed.
